# H_2_O_2_-Responsive Nanoparticle Based on the Supramolecular Self-Assemble of Cyclodextrin

**DOI:** 10.3389/fphar.2018.00552

**Published:** 2018-05-28

**Authors:** Zhenqiang Dong, Yang Kang, Qijuan Yuan, Manli Luo, Zhipeng Gu

**Affiliations:** ^1^Key Laboratory of Sensing Technology and Biomedical Instrument of Guangdong Province, School of Engineering, Sun Yat-sen University, Guangzhou, China; ^2^College of Chemical and Material Engineering, Quzhou University, Quzhou, China; ^3^Key Laboratory of Mountain Ecological Restoration and Bioresource Utilization, Chengdu Institute of Biology, Chinese Academy of Sciences, Chengdu, China; ^4^Guangdong Provincial Key Laboratory of Malignant Tumor Epigenetics and Gene Regulation, Sun Yat-sen Memorial Hospital, Sun Yat-sen University, Guangzhou, China

**Keywords:** responsive, cyclodextrin, self-assemble, nanoparticle, BSA

## Abstract

Designing stimuli responsive, controllable and biocompatible multifunctional nanoparticles is an important progress in the current quest for drug delivery systems. Herein, we devoted to developing a β-cyclodextrin (β-CD) based drug delivery nanoparticles (NPs) that release Bovine serum albumin (BSA) via glucose-responsive gate. The design involves synthesis of sodium alginate with β-CD modified (Alg-β-CD) and methoxypolyethylene glycol (mPEG-Fc) containing ferrocene (Fc) uncharged end-capping. When α-cyclodextrin (α-CD) was added with these two segments, the stable non-covalent supramolecular structure of Alg-β-CD/mPEG-Fc/α-CD can be self-assembled into NPs in aqueous solution. BSA loaded Alg-β-CD/mPEG-Fc/α-CD also has been prepared. Interestingly, these supramolecular Alg-β-CD/mPEG-Fc/α-CD/BSA NPs showed uniform sphere structure and constant BSA loading content. Also, this new kind of NPs can disassemble in the present of hydrogen peroxide (H_2_O_2_). Since glucose oxidase (GOD) can oxidize glucose and produce H_2_O_2_, so this kind of polymeric NPs can also have glucose responsive behavior in the GOD containing environment. Developed functional Alg-β-CD/mPEG-Fc/α-CD might be a promising drug delivery strategy for diabetes or immunotherapy with more efficiency.

## Introduction

For the past decades, stimuli responsive nanoparticles (NPs) based delivery technique have emerged as a promising strategy for controllable drug delivery, especially for protein delivery due to ingenious design (Tao et al., 2013; Wu et al., 2014; Kuang et al., 2016; Ding et al., 2017; Kang et al., 2017b). As smart vehicles, these ideal NPs can undergo reversible physical or chemical changes to control drug release in response to external stimuli such as pH, light, temperature, redox and molecules (Kang et al., 2015; Tao et al., 2016, 2017a,b; Rosenblum et al., 2018; Zhu et al., 2018). For cancer therapy, diverse intracellular and extracellular endogenous stimuli within tumor microenvironment, such as low pH value, hypoxia, enzymes and reactive oxygen species (ROS) have been widely investigated in stimuli responsive NPs and explored as the stimuli to selectively trigger drug control release or transfer prodrug systems into active form (Chen et al., 2015; Ruan et al., 2015, 2016; Liao et al., 2016; Wang et al., 2017b). As an important hallmark, it should be noticed that various studies have demonstrated that hydrogen peroxide (H_2_O_2_) could be used as a second messenger in intracellular signaling cascades and would be generated in multiple skin lesions, initiating aging cells and the tumoral tissue (Miao et al., 2015). In this regard, H_2_O_2_-responsive NPs would be a promising therapeutic strategy for the treatment of specific disease.

At present, block copolymer approaches for H_2_O_2_-responsive NPs has been widely used and rapidly developed. For instance, a tyrosol incorporated copolyoxalate (TPOX) NPs have been developed by Kim et al. (2017), which demonstrated that TPOX NPs released entrapped nile red through the degradation under H_2_O_2_ stimulation and could be used as H_2_O_2_-responsive therapeutic NPs for the treatment and inflammatory. Recently, Gu group have designed a glucose-responsive mechanism directly utilizing H_2_O_2_-sensitive polymeric vesicles by using polyethylene glycol (PEG) and phenylboronic ester (PBE)-conjugated polyserine for smart insulin delivery (Hu et al., 2017). Apart from stimuli-responsive polymeric NPs via cleavable covalent bonds to control their properties, supramolecular based NPs via non-covalent bonds have also been widely studied due to that functional ligands and environmental responsive bonds can be conveniently incorporated through host-guest interaction, π–πinteraction, hydrogen bonding interaction and so on (Wang et al., 2015, 2017a). Cyclodextrin (CD), as a most common case of supermolecules, which has been demonstrated that can bind some specific hydrophobic guest molecules into its cavities to construct stable host-guest complex (Yuan et al., 2013). CDs are also considered as a good choice for drug delivery strategy due to their adjustable water solubility, good biocompatibility, and non-toxicity toward biological systems (Kang et al., 2014). In the previous studies, various stimuli-responsive CDs NPs based on host-guest interactions between CDs and different guest molecules [such as azobenzene, ferrocene (Fc), and adamantine] have been widely developed. For instance, Guo and Lei (2015) have developed a kind of redox-responsive cationic supramolecular polymers based on the host-guest interaction between β-CD and Fc, which indicated the potential application for gene nanocarrier. To conduct the redox-stimuli in an effect way, Kang et al. (2017a) have designed a kind of dual redox-responsive micelles which fabricated by methoxypolyethylene glycol conjugated β-CD (mPEG-β-CD) and Fc conjugated camptothecin (Fc-SS-CPT) segments for achieving dual ROS and glutathione responsive. It is reasonable to design H_2_O_2_-sensitive NPs based on CD, which can load and controllable releasing drugs for treatment of specific disease.

**FIGURE 1:**
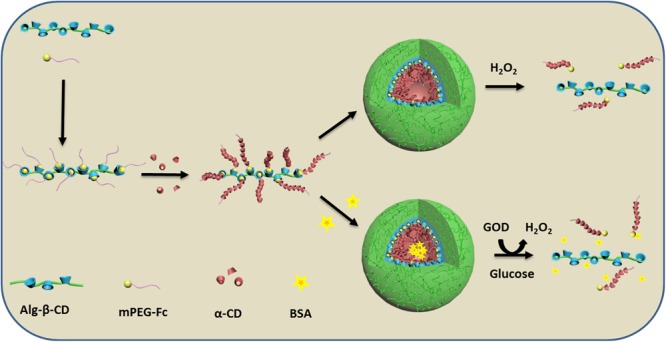
Schematic illustration of the formation of Alg-β-CD/mPEG-Fc/α-CD nanoparticles and their enzyme encapsulation and controlled release behavior.

In this paper, a novel oxidation responsive rod-coil polymeric NPs have been prepared using water as the self-assemble solvent (**Figure 1**) Firstly, sodium alginate with β-CD end-decoration (Alg-β-CD) and mPEG conjugated with Fc (mPEG-Fc) were designed and synthesized, and then the self-assembly behavior of them in water was studied in detail. This supramolecular copolymer grows up to form comb shaped polymer in aqueous solution by the terminal host-guest inclusion. When adding certain amount of α-CD to this solution, the PEG will form inclusion complex with α-CD to form the rod section while sodium alginate form the coil section. This kind of structure will self-assemble into rod-coil NPs in water and load Bovine serum albumin (BSA). This new kind of NPs can disassemble in the present of H_2_O_2_. Since glucose oxidase (GOD) can oxidize glucose and produce H_2_O_2_, so this kind of polymeric nanosphere can also have glucose responsive behavior in the GOD containing environment. This will make it very promising to use in biomedical application field in body environment.

## Materials and Methods

### Materials and Reagents

β-cyclodextrin (β-CD), α-Cyclodextrin (α-CD) was purchased from Sigma and dried in a vacuum before use. Sodium alginate (Alg) was purchased from Tianjin Yuanhang Chemicals Co., Ltd., China. Methoxypolyethylene glycol (mPEG, Mw = 2000) was purchased from Shanghai Jingchunshiye Co., Ltd., China. Glucose oxidase was purchased from Aspergillus Niger (GOD, lyophilized powder protein, Mw = 186 000, catalase <4%). BSA was purchased from Aoke Biological Co., Ltd., China. Carboxyferrocene (Fc-COOH), morpholinoethanesulfonic acid (MES), dicyclohexylcarbodiimide (DCC), 4-(dimethylamino) pyridine (DMAP), *N*-hydroxysuccinimide (NHS), 1-ethyl-3-[3-(dimethylamino)propyl]carbodiimide hydrochloride (EDCI), and other reagents were purchased from Chengdu Kelong Co., Ltd., China.

### Synthesis of Alg-β-CD and mPEG-Fc

**FIGURE 2:**
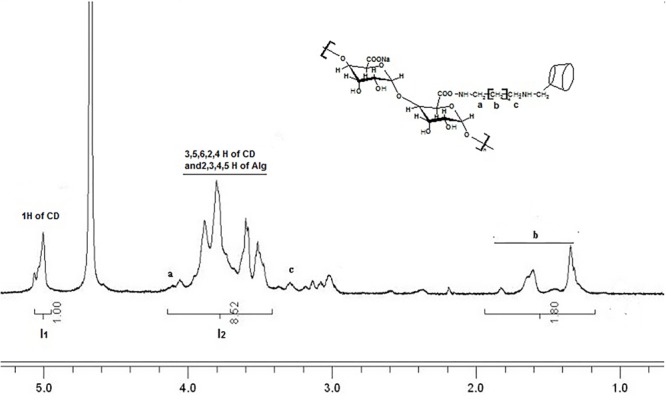
^1^HNMR spectrum of sodium alginate with β-CD (Alg-β-CD) in D_2_O. a–f mean the different H of the molecule structure.

Sodium alginate with β-CD was synthesized in two steps with slight modification. Firstly, the mono-6-(*p*-tolylsulfonyl)-β-cyclodextrin (6-β-CD-OTs) and mono-6-deoxy-6-hexamethylenediamine-β-CD (6-β-CD-HDA) were synthesized according to the method reported in the previous study (Quan et al., 2010). Briefly, β-CD was grafted onto the sodium alginate backbone via amido link condensation reaction. 1% sodium alginate solution (w/v) was prepared in MES buffer solution (0.1 M) and NaCl (0.5 M), and the pH was adjusted to 6.0. After 29 mg NHS and 95 mg EDC (molar ration of EDC: NHS: COO- = 1: 0.5: 1) were added to 40 mL above alginate solution, the solution was then agitated for 30 min to obtain a homogeneous solution followed by the addition of 10 mL 3.08 % 6-β-CD-HDA MES solution (w/v). The reaction was then continuing at 4°C for 24 h. Alg-β-CD was obtained after the resulting mixture dialyzed against pure water and then freeze dried. The degree of β-CD substitution (DS) to alginate was calculated according to the followed equation:

$$\begin{array}{c} \frac{I_{1}}{I_{2}-6I_{1}-\frac{2}{7}I_{2}}=\frac{7}{4}\mathrm{DS} \end{array}$$

where *I*_1_ is the digital integration of anomeric protons of β-CD, *I*_2_ is the total digital integration of 3.4–4.2 ppm. The final degree of substitution (DS) of Alg-β-CD was 25.6 % by this calculated method. ^1^HNMR (400 MHz, D_2_O) were shown in **Figure 2**.

**FIGURE 3:**
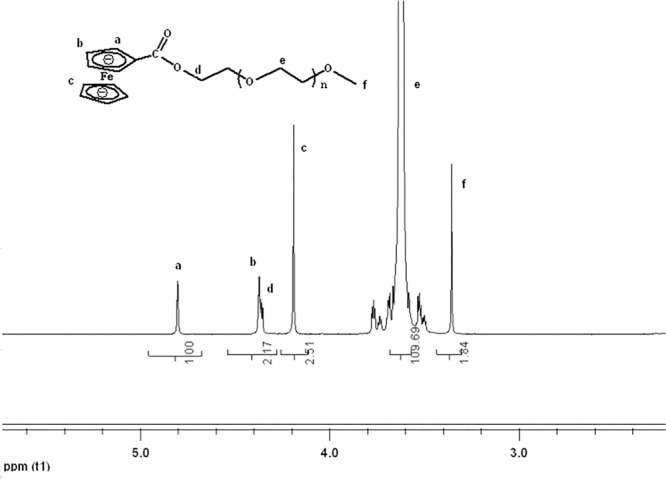
^1^H NMR spectrum of mPEG-Fc in CDCl_3_. a-f mean the different H of the molecule structure.

mPEG-Fc was synthesized with one pot method. DMAP (0.183 g) and DCC (1.12 g) were added successively to a solution of mPEG (3.0 g) and carboxyferrocene (0.69 g) in dry chloroform (CHCl_3_, 100 mL) for 24 h at room temperature. After removing dicyclohexylurea (DCU) by filtration, the filtrate was concentrated in a vaccum and purified by dissolving in deionized water and extracting with diethyl ether four times. Subsequently, the product was further purified by using gel column with methanol and water as the solvent to remove the unreacted carboxyferrocene and catalyst residue. Finally, the dark red product was concentrated in a vaccum and dried in the vacuum oven for 2 days. The final DS of PEG-Fc used was 95.4% which was calculated though the integral ratio between H_d_ and H_f_. ^1^HNMR (400 MHz, CDCl_3_) were shown in **Figure 3**.

### Preparation of Alg-β-CD/mPEG-Fc/α-CD and Alg-β-CD/PEG-Fc/α-CD/BSA NPs

PEG-Fc (6.6 mg) and Alg-β-CD (8.4 mg) were added to 3 mL deionized water and kept stirring for 5 h to get a transparent solution. Then α-CD (0.2 g) was added to the above solution and continued to stir over night. For BSA loading, 1.5 mg BSA was added with stirring for 10 min before the addition of α-CD. The solution changes from transparent to turbid gradually indicating the formation of the Alg-β-CD/PEG-Fc/α-CD and Alg-β-CD/PEG-Fc/α-CD/BSA NPs.

### Characterization of the Alg-β-CD/mPEG-Fc/α-CD and Alg-β-CD/mPEG-Fc/α-CD/BSA NPs

The product was characterized by ^1^H–^1^H (2D) NOESY NMR spectroscopy which recorded on an Advance Bruker 600 NMR spectrometer. The transmission electron microscopy (TEM) images were taken by using a JEOL JEM-100CX instrument operating at an accelerating voltage of 80 kV. Each sample were prepared and imaged by Atomic force microscopy (AFM) in tapping mode with a Nanoscope IIIa Digital Instrument. X-ray powder diffraction (XRD) patterns of the samples were recorded by using Cu-Kα irradiation source with X’ Pert MPD (20 kV; 35 mA; 2°theta/min) to determine the crystalline structures.

### Self-Assembly NPs Encapsulation and Leakage of BSA

Self-assembly NPs encapsulated BSA were prepared and then their encapsulation efficiency was determined as follows. The BSA loaded NPs (0.5 mg BSA, 0.5%) was centrifuged for 20 min at 12,000 rpm to completely separate the free and encapsulated BSA and the supernatant was analyzed by determined by UV spectrophotometry. The BSA encapsulation efficiency was calculated as follows (1):

$$\begin{array}{c} \mathrm{Bovine}\;\mathrm{serum}\;\mathrm{albumin}\;\mathrm{encapsulation}\;\mathrm{efficiency}\;(\%)\;=\frac{\mathrm{mass}\;\mathrm{of}\;\mathrm{BSA}\;\mathrm{used}\;\mathrm{in}\;\mathrm{formulation}-\mathrm{mass}\;\mathrm{of}\;\mathrm{free}\;\mathrm{BSA}}{\mathrm{mass}\;\mathrm{of}\;\mathrm{BSA}\;\mathrm{used}\;\mathrm{in}\;\mathrm{formulation}}\times 100 \end{array}$$

Then the NPs were continuing stored in aqueous solution for 48 h to evaluate leakage of BSA. The mixture was again separated by centrifugation and the supernatant was analyzed for determination of BSA leakage efficiency as above method (2):

$$\begin{array}{c} \mathrm{Bovine}\;\mathrm{serum}\;\mathrm{albumin}\;\mathrm{leakage}\;\mathrm{efficiency}\;(\%)\;=\frac{\mathrm{mass}\;\mathrm{of}\;\mathrm{BSA}\;\mathrm{in}\;\mathrm{the}\;\mathrm{supernatant}}{\mathrm{mass}\;\mathrm{of}\;\mathrm{BSA}\;\mathrm{encapsulated}}\times 100 \end{array}$$

### *In Vitro* Release Studies

The Alg-β-CD/PEG-Fc/α-CD self-assembly NPs encapsulated BSA was prepared and centrifuged for *in vitro* BSA release study (12,000 rpm, 20 min). The lower sediment was added 12 mL 0.1 M glucose solution and 10 mg GOD and kept stirring for 10 min. The solution was placed in a constant temperature incubator and kept the temperature at 37°C during the whole release process. 2 mL solution had been taken out and centrifugation at 12,000 rpm for 20 min every 6 h. The amount of BSA released in the supernate was collected and determined by UV spectrometry at specified time. The BSA release was quantified when the standard curve was plotted using a standard BSA solution.

### *In Vitro* Cell Studies

Methyl tetrazolium (MTT) assay was carried out on murine colon carcinoma cells (CT26) as the cell line for biocompatibility evaluation of the self-assembly NPs. 5 × 10^3^ cells were culture for 24 h to attach in 96-well plates. Then the cells were treated with fresh medium containing 1 mg/mL Alg-β-CD/mPEG-Fc/α-CD NPs and Alg-β-CD/mPEG-Fc/α-CD/BSA NPs. The cell viability was measured after incubation for desired time duration (48 h). The cells without treatment were used as the control. Each group was repeated in triplicate.

Further, confocal laser scanning microscopy (CLSM) was used for monitoring the *in vitro* endocytosis. Briefly, self-assembly NPs encapsulated Dil were prepared and incubated with CT26 cells at 37°C for 4 h. After incubation, all samples were stained with LysoTracker Green (Life Technologies, Carlsbad, CA, United States) and 10 μg/mL of Hoechst 33342 (Life Technologies, Carlsbad, CA, United States).

### Statistical Analysis

All the results obtained in this study were expressed as mean ± standard deviation unless otherwise noted. Comparisons between two groups were made using two-tailed student’s *t*-test and between multiple groups were made using one-way analysis of variance (ANOVA). Statistical analyses were performed using SPSS 21.0 and considered statistically significance when ^∗^*p* < 0.05.

## Results and Discussion

**FIGURE 4:**
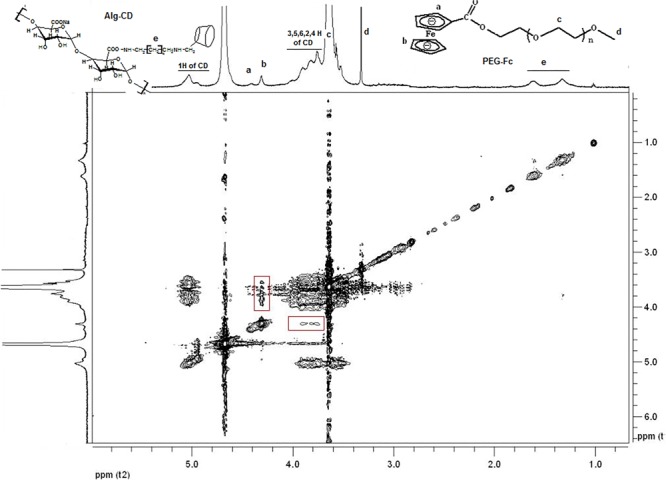
2D-NOESY-NMR spectrum of mixture of Alg-β-CD and PEG-Fc inclusion complex in D_2_O. a-f mean the different H of the molecule structure.

According to the previous, it has demonstrated that inclusion complexes would form when β-CD interact with Fc group (Tomatsu et al., 2010). Further, the formation of complexation between the Fc groups on mPEG-Fc and β-CD groups on Alg-β-CD could be observed by using 2D NMR NOESY spectra. As shown in **Figure 4**, the peaks of β-CD group were in accordance with the resonance of the Fc groups could be found in the 2D NOESY spectrum of Alg-β-CD/PEG-Fc, which indicating that the Fc groups threaded through the cavity of β-CD to form an inclusion complex.

**FIGURE 5:**
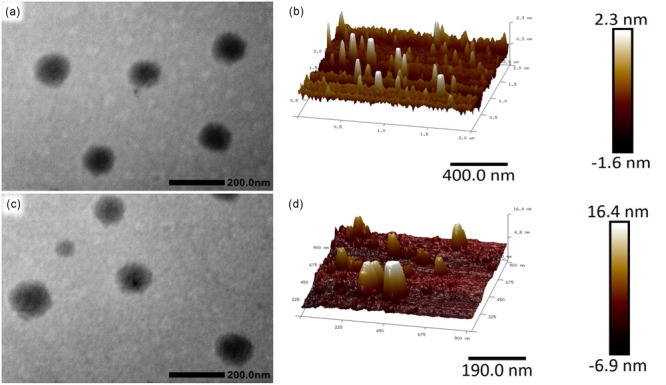
**(a)** Transmission electron microscopy (TEM) micrograph of Alg-β-CD/PEG-Fc/α-CD NPs; **(b)** Atomic force microscopy (AFM) macrograph of Alg-β-CD/PEG-Fc/α-CD NPs; **(c)** TEM micrograph of Alg-β-CD/PEG-Fc/α-CD NPs entrapped Bovine serum albumin (BSA); **(d)** AFM macrograph of Alg-β-CD/PEG-Fc/α-CD NPs entrapped BSA.

The resulting Alg-β-CD/PEG-Fc was hydrophilic copolymers, which could form transparency solution in water. By adding α-CD to the aqueous solution of Alg-β-CD/PEG-Fc at room temperature, the solution gradually became turbid gradually when the aggregates were formed. The morphology of these aggregates has been observed by TEM and atomic force microscope (AFM). TEM micrographs of **Figure 5a** revealed that the Alg-β-CD/PEG-Fc/α-CD aggregates appeared round line shapes and their diameters were about 100 nm. In the AFM micrographs of **Figure 5c**, Alg-β-CD/PEG-Fc/α-CD self-assembly NPs also showed rounded line shapes with a horizontal length about 100 nm. Particularly, it is noted that most height of the aggregates (<5 nm) was about 20 times smaller than horizontal length. The main reason for such size differences between horizontal and vertical direction might be the collapsing of this NPs. Further, the height of NPs increased about threefold when BSA was encapsulated, which demonstrated that the BSA has been successfully encapsulated into the Alg-β-CD/PEG-Fc/α-CD NPs (**Figures 5b,d**).

**FIGURE 6:**
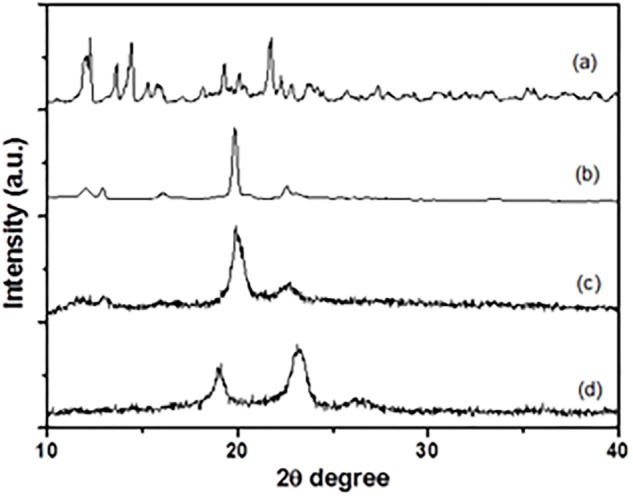
X-ray diffraction patterns of **(a)** α-CD, **(b)** PEG/α-CD inclusion complex, **(c)** Alg-β-CD/PEG-Fc/α-CD particles, **(d)** Alg-β-CD/PEG-Fc.

The XRD patterns of the α-CD (**Figure 6a**), PEG/α-CD inclusion complex (**Figure 6b**), Alg-β-CD/PEG-Fc/α-CD (**Figure 6c**), and Alg-β-CD/PEG-Fc (**Figure 6d**) has been shown in **Figure 6**. As an important index to evaluate the inclusion structures formation of CD based complex. The pattern of Alg-β-CD/PEG-Fc/α-CD showed in the figure was differs from that of free Alg-β-CD/PEG-Fc or α-CD. It was also found that the homo-PEG crystalline peaks (2 theta = 19.2° and 23.3°) and α-CD crystalline peak (2 theta = 21.5°) were absent. According to the previous study, the peak at 2 theta = 19.9° is a typical peak of PEG-α-CD channel-type crystallites that demonstrated the inclusion structures of Alg-β-CD/PEG-Fc/α-CD contained PEG-α-CD (Lautens et al., 1990). Meanwhile, the NPs would form an inner rod-like PEG-α-CD inclusion block surrounded by a protonated coil-like Alg shell when insoluble PEG-α-CD inclusion blocks formed and water became a selective solvent for the Alg-β-CD/PEG-Fc/α-CD. This rod-like block in rod-coil system preferred parallel packing which may result in the formation of hollow NPs promoted by efficient space-filling has been investigated (Jenekhe and Chen, 1999).

**FIGURE 7:**
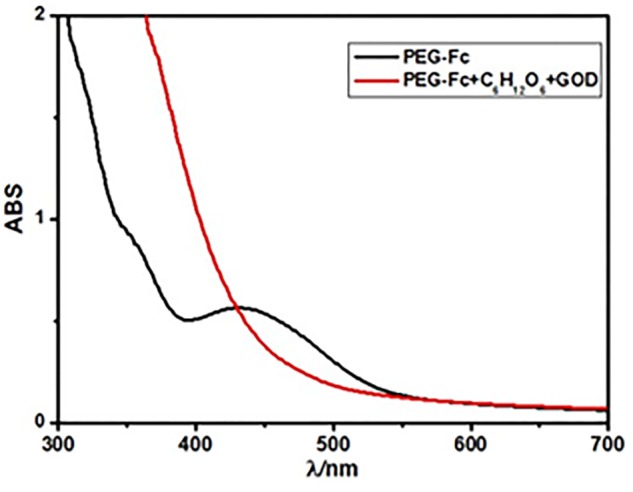
UV-vis spectra of the PEG-Fc in 12 mL 0.1 M glucose before and after 10 mg glucose oxidase (GOD) was added after 12 h.

As mentioned in the literature and our previous study, the Alg-β-CD/PEG-Fc/α-CD self-assemble NPs were connected by the Fc-β-CD inclusions. These NPs could release BSA through their collapse after the dissociation of Fc-β-CD. To achieve this goal, the oxidization Fc to Fc^+^ is an efficacy way while β-CD could not form complex with Fc derivatives in their oxidized state (Matsue et al., 1985). To get a clear vision, hydrogen peroxide (H_2_O_2_) was chosen as the oxidant in this study. After the addition of H_2_O_2_ (1 mL, 30%, H_2_O_2_ solution) to Alg-β-CD/PEG-Fc/α-CD hollow NPs (10 mL, 1%), the system became unstable and produced polyrotaxane precipitate in 5 min. The possible mechanism of this phenomenon can be seen in **Figure 7**. The addition of H_2_O_2_ dissociated Fc-β-CD inclusion complex and leaving the rod-like hydrophobic PEG-Fc/α-CD part precipitated out of water.

Nowadays, one of the most widely used applications of the stimuli-responsive NPs is in the on demand drug-release field (Tao et al., 2015). It requires the micelle system release the aim drugs gradually to achieve the long-term drug action. Since H_2_O_2_ could be produce in a rather sustained way through oxidization of glucose by glucose oxidase (GOD) so that can oxidize Fc to its oxidation state gradually. **Figure 7** shows the UV-vis spectra of the PEG-Fc in 0.1 M glucose before and after 10 mg GOD was added. After adding GOD for 12 h, the absorption peak around 450 nm due to Fc groups d–d transition peak (in reduced form) almost disappeared indicating that the Fc groups in the PEG-Fc could be oxidized to ferrocenium as anticipated (Yamamoto et al., 2000). Tan et al. (2012) have been developed the glucose sensitive hydrogel using the GOD and glucose interaction to produce hydrogen peroxide to disintegrate the ICs of Fc and β-CD. Therefore, it is possible to get glucose sensitive Alg-β-CD/PEG-Fc/α-CD micelles by adding GOD to the Alg-β-CD/PEG-Fc/α-CD system.

**FIGURE 8:**
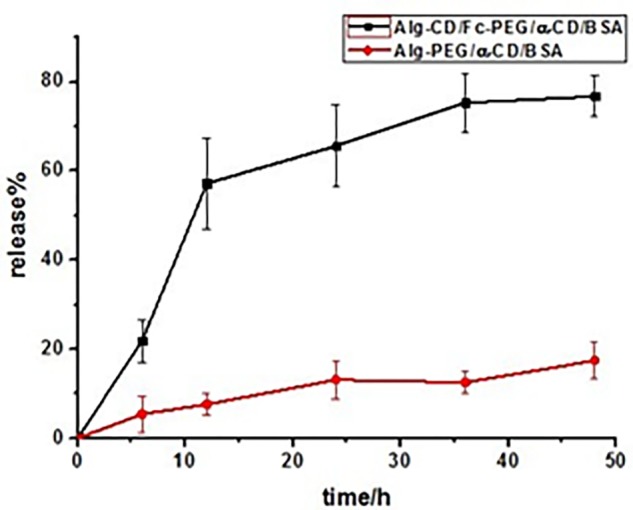
Bovine serum albumin release from Alg-β-CD/PEG-Fc/α-CD/BSA and Alg-PEG/α-CD/BSA nanoparticles at 37°C in 12 mL 0.1 M glucose and 10 mg GOD containing solution.

Otherwise, serum albumins, as a major soluble protein, have got much attention and been widely used due to their physiological functions. It has been applied in the nanomedicine field because of they serve as a depot protein and as a transport protein for a variety of compounds. BSA has been one of the most extensively member used as a protein drug model, particularly because of its structural homology with human serum albumin (Papadopoulou et al., 2005). The incorporation of BSA into Alg-β-CD/PEG-Fc/α-CD particles had been studied. The encapsulation efficiency and leakage efficiency was 71.4 and 6.6%, respectively. The *in vitro* release profile of BSA from Alg-β-CD/PEG-Fc/α-CD/BSA and Alg-PEG/α-CD/BSA particles was presented in **Figure 8**. In Alg-β-CD/PEG-Fc/α-CD/BSA particles group, the BSA appeared to be released in a continuous way. After 48 h, about 80% of BSA had been released. While as a counterpart, the Alg-PEG/α-CD/BSA group (BSA encapsulation efficiency was 66.7%), under the same condition, there was less than 20% of BSA had been released which is much less than the Alg-β-CD/PEG-Fc/α-CD/BSA group. This result further confirmed that in glucose and GOD containing environment the Alg-β-CD/PEG-Fc/α-CD/BSA particle achieved BSA delayed release behavior through the β-CD-Fc inclusion complex as redox trigger.

**FIGURE 9:**
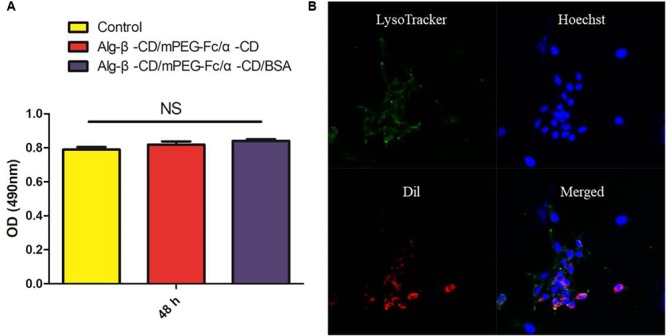
**(A)** The *in vitro* cytotoxicity of Alg-β-CD/PEG-Fc/α-CD NPs with and without BSA; **(B)** The cellular uptake of Alg-β-CD/PEG-Fc/α-CD NPs.

The biocompatibility of NPs is of critical importance to their eventual success (Tao et al., 2014). As shown in **Figure 9A**, the results demonstrate the non-toxic of Alg-β-CD/PEG-Fc/α-CD and Alg-β-CD/PEG-Fc/α-CD/BSA NPs, which could be used as the biocompatible drug delivery system. The performance of NPs cellular internalization through the observation of NPs encapsulated Dil interaction with labeling CT26 cells (**Figure 9B**). The results indicated that large numbers of Alg-β-CD/PEG-Fc/α-CD NPs entered the cells after 4 h and some of them began to enter into cell nucleus.

## Conclusion

In summary, Alg-β-CD/PEG-Fc/α-CD rod-coil hollow NPs have been prepared through the supramolecular self-assemble. The rod-part PEG-α-CD and the coil-part Alg were connected through β-CD-Fc inclusion complex. The BSA enzyme was successfully encapsulated in Alg-β-CD/PEG-Fc/α-CD hollow sphere in aqueous solution. These Alg-β-CD/PEG-Fc/α-CD hollow NPs showed good H_2_O_2_ sensitivity. Further study showed that this nanoparticle encapsuled BSA can achieve BSA delayed drug-release behavior in the glucose containing environment at the existence of GOD. Therefore, this study exhibits that using the host-guest interactions would be a general, useful and effective way for fabricating stimuli-responsive drug delivery NPs. Furthermore, the glucose mediated H_2_O_2_ sensitive behavior of the Alg-β-CD/PEG-Fc/α-CD NPs may benefit its application in more and more fields such as enzyme immobilization, drug delivery and micro-reactors.

## Author Contributions

ZG designed experiments, supervised the experiments, and revised and finalized the manuscript. ZD and YK performed the experiments, analyzed the data, and prepared the figures and the manuscript. QY performed the cell culture. ML contributed to the results discussion and paper writing. All authors reviewed and approved the final paper.

## Conflict of Interest Statement

The authors declare that the research was conducted in the absence of any commercial or financial relationships that could be construed as a potential conflict of interest.
